# Supplementary material for: Saving less in China facilitates global CO2 mitigation
Source: Nat Commun. 2020 Mar 13;11:1358. doi: 10.1038/s41467-020-15175-2 (PMC7070072; doi:10.1038/s41467-020-15175-2)
Supplement: Supplementary file 1 — Supplementary Information [file 41467_2020_15175_MOESM1_ESM.pdf]

Supplementary Information

Saving less in China facilitates global CO_2 mitigation

Lin et al.

Supplementary Figures

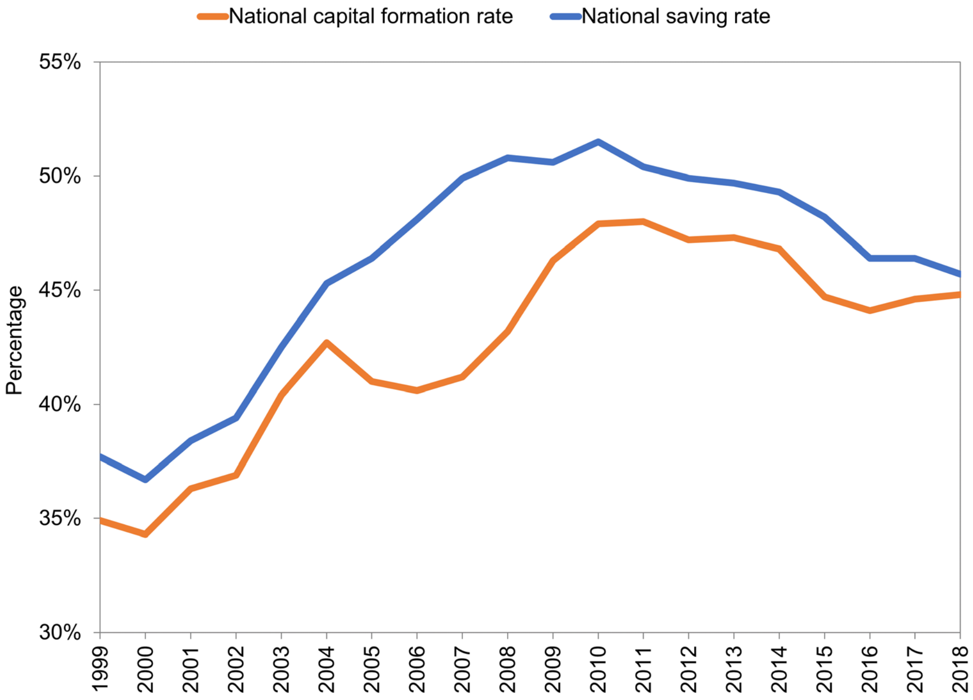

Supplementary Figure 1. National capital formation rates and national saving rates during 1999-2018[1]. The changes of the saving rates and capital formation rates in China were synchronous in most years.

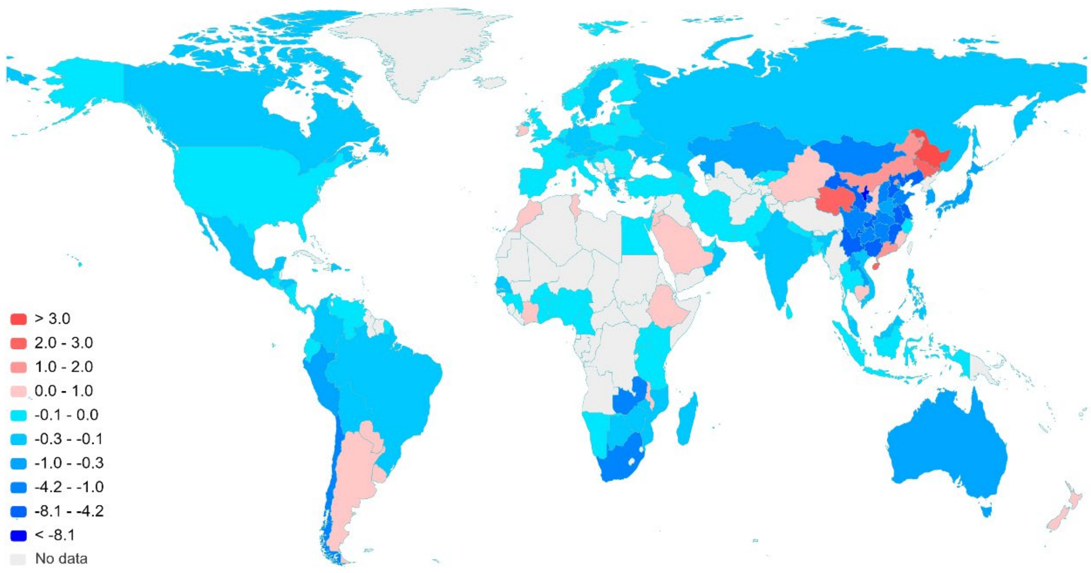

Supplementary Figure 2. Percentage portions of CO_2 emission changes of nations and Chinese regions (%), with conditions of saving rates of all Chinese regions decreasing by 15 percentage points simultaneously and emission intensity as of 2012 (Scenario 1).

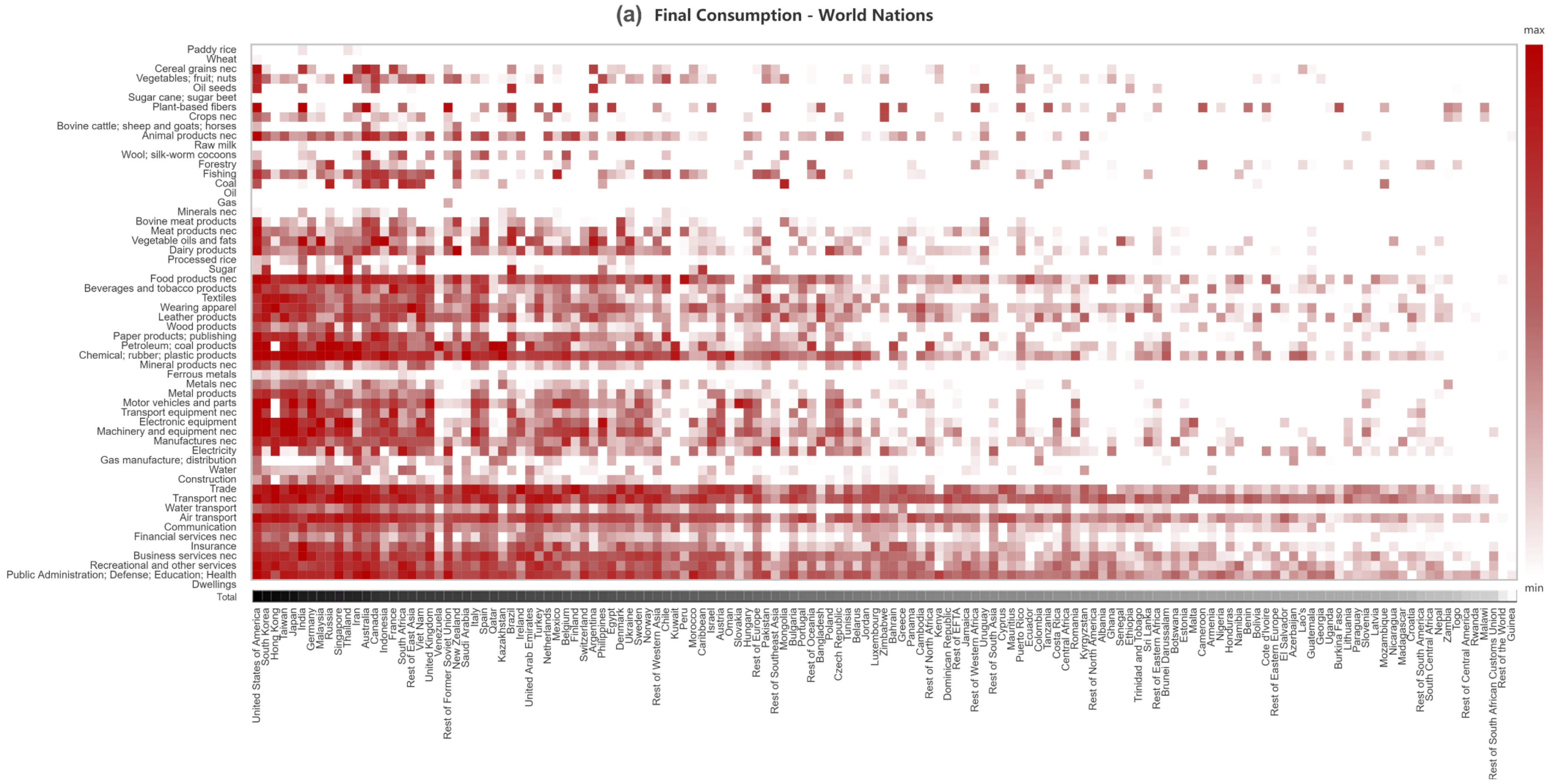
(a) Final Consumption - World Nations
max
min
Paddy rice
Wheat
Cereal grains nec
Vegetables; fruit; nuts
Oil seeds
Sugar cane; sugar beet
Plant-based fibers
Crops nec
Bovine cattle; sheep and goats; horses
Animal products nec
Raw milk
Wool; silk-worm cocoons
Forestry
Fishing
Coal
Oil
Gas
Minerals nec
Bovine meat products
Meat products nec
Vegetable oils and fats
Dairy products
Processed rice
Sugar
Food products nec
Beverages and tobacco products
Textiles
Wearing apparel
Leather products
Wood products
Paper products; publishing
Petroleum; coal products
Chemical; rubber; plastic products
Mineral products nec
Ferrous metals
Metals nec
Metal products
Motor vehicles and parts
Transport equipment nec
Electronic equipment
Machinery and equipment nec
Manufactures nec
Electricity
Gas manufacture; distribution
Water
Construction
Trade
Transport nec
Water transport
Air transport
Communication
Financial services nec
Insurance
Business services nec
Recreational and other services
Public Administration; Defense; Education; Health
Dwellings
Total

(b) Capital Formation - World Nations

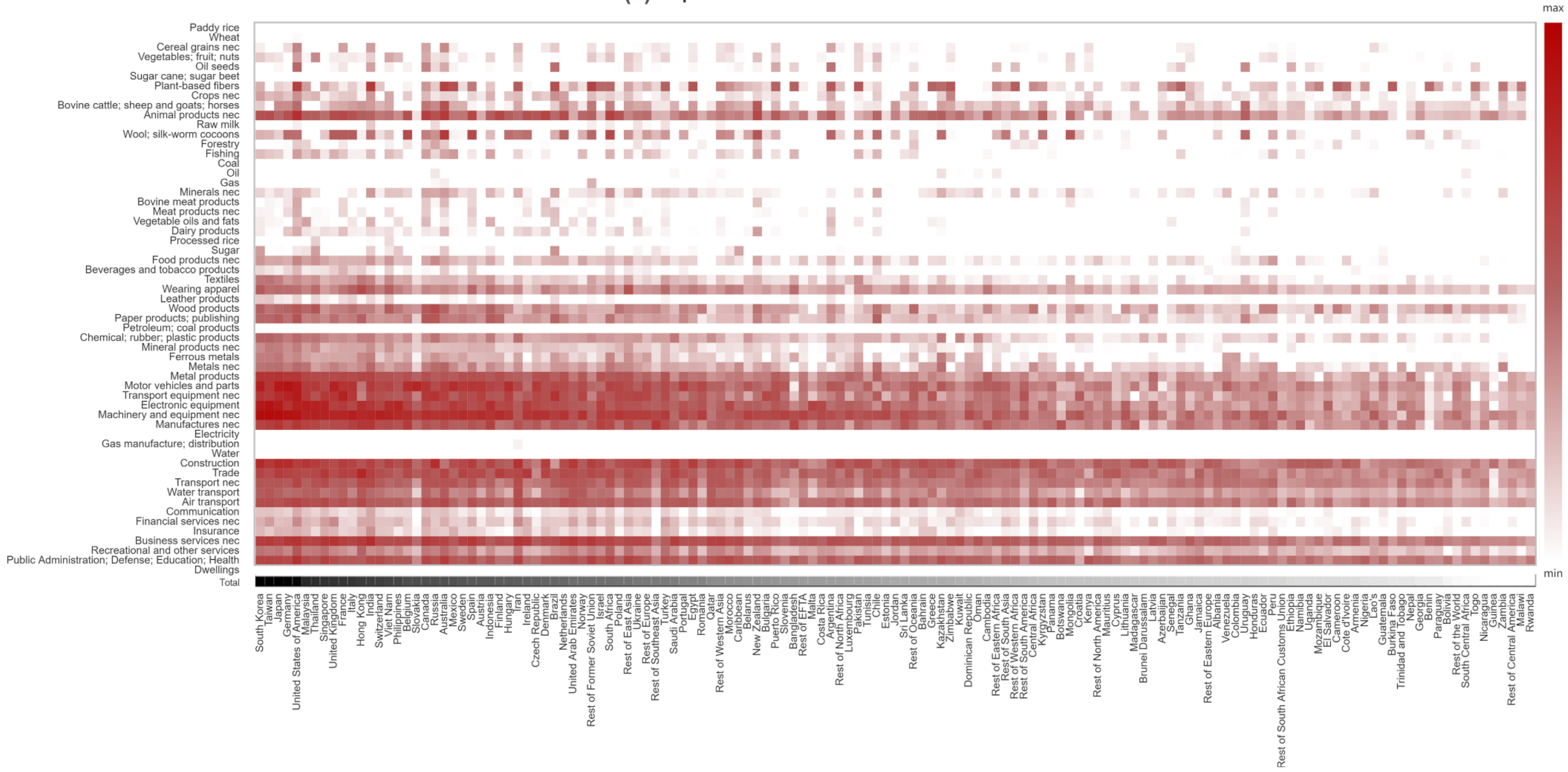

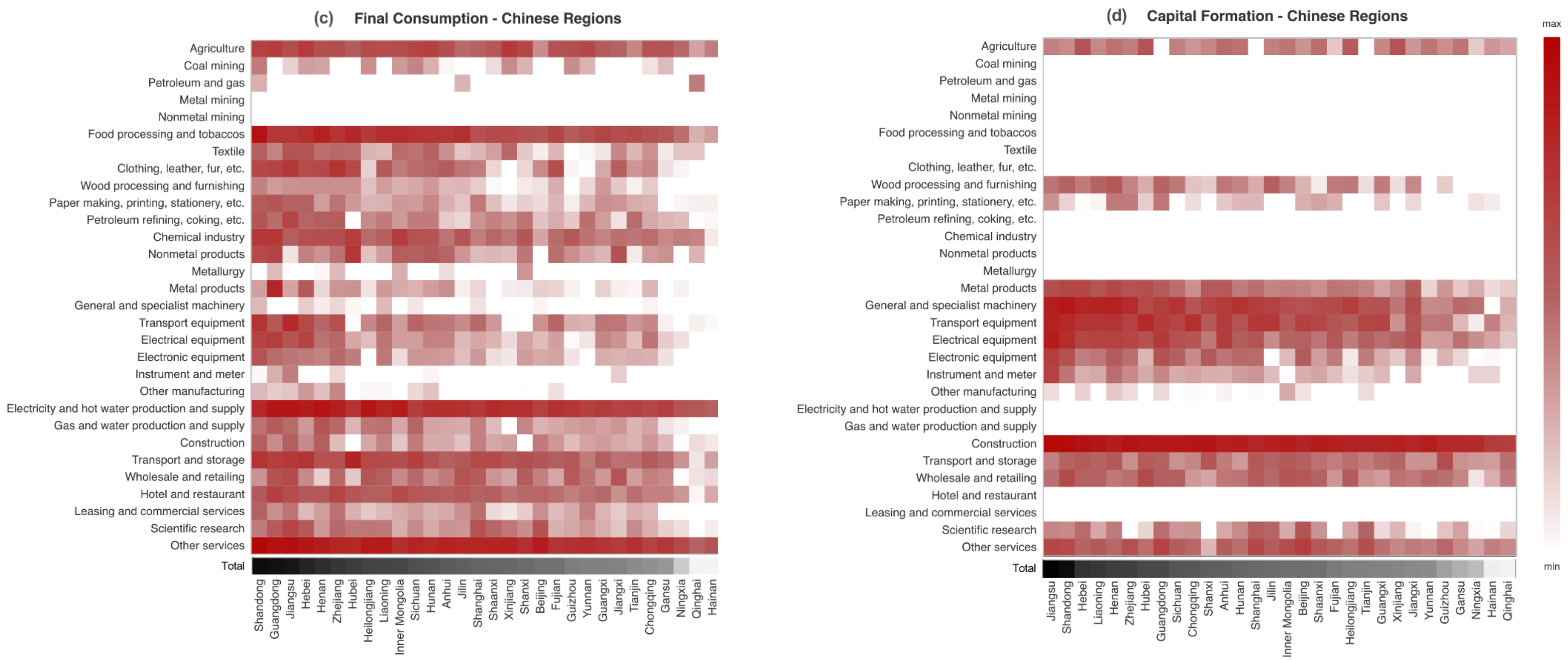

Supplementary Figure 3. Global CO_2 emissions induced by unitary final consumption or capital formation in China. Each grid in Supplementary Figures 3a and 3c indicates global CO_2 emissions induced by China's unitary final consumption of products from each sector of nations (a) and Chinese regions (c). Each grid in Supplementary Figures 3b and 3d indicates global CO_2 emissions induced by China's unitary capital formation of products from each sector of nations (b) and Chinese regions (d). The black/white bars at the bottom show the total global CO_2 emissions caused by China's unitary final consumption or capital formation of nations or Chinese regions. A darker colour indicates larger global CO_2 emissions.

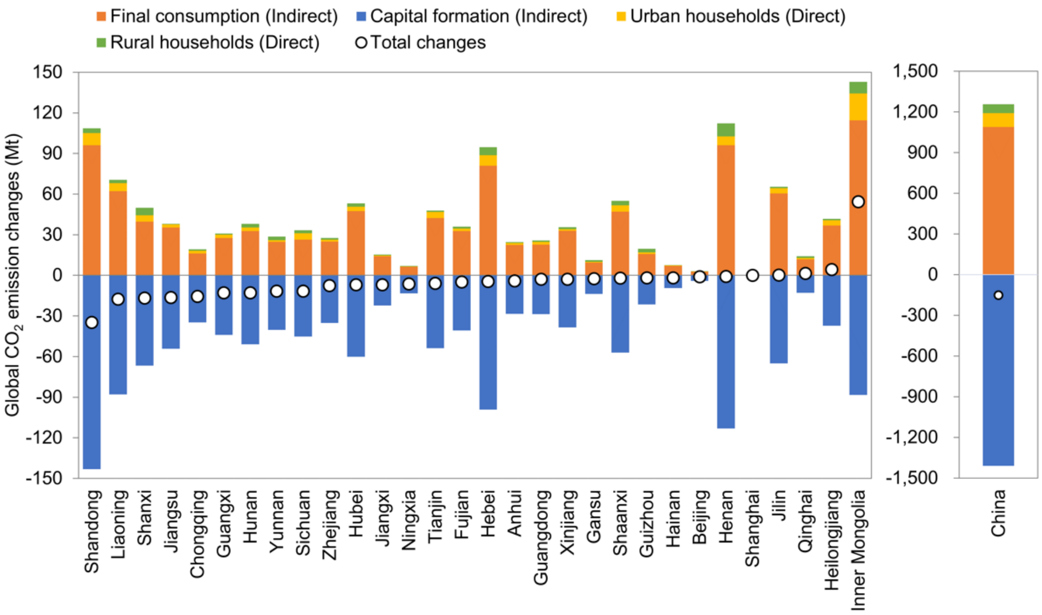

Supplementary Figure 4. Global CO_2 emission changes caused by reducing the saving rate of each Chinese region to Shanghai's saving rate in 2012 (37%).

(a)

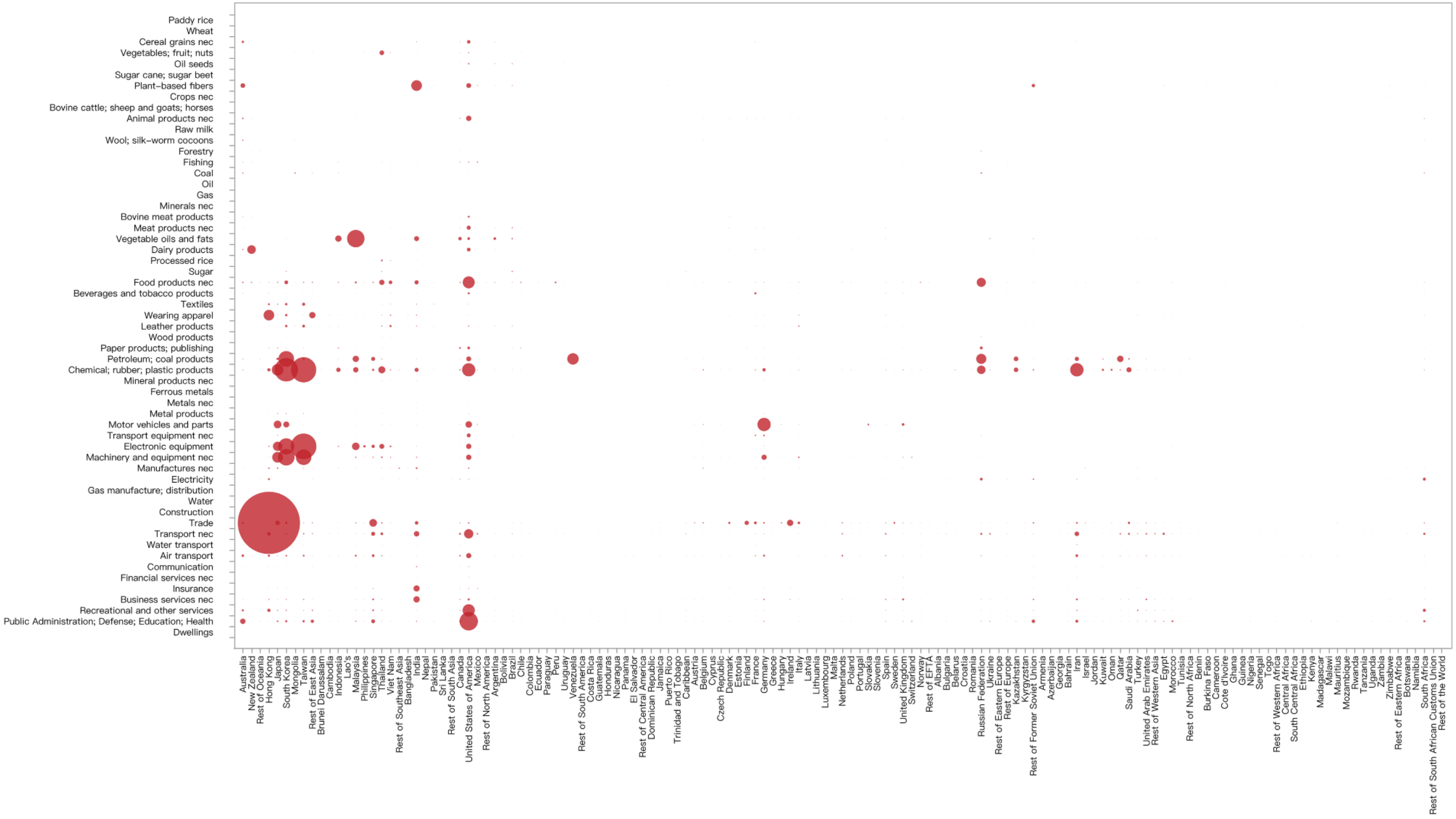

Paddy rice
Wheat
Cereal grains nec
Vegetables; fruit; nuts
Oil seeds
Sugar cane; sugar beet
Plant−based fibers
Crops nec
Bovine cattle; sheep and goats; horses
Animal products nec
Raw milk
Wool; silk−worm cocoons
Forestry
Fishing
Coal
Oil
Gas
Minerals nec
Bovine meat products
Meat products nec
Vegetable oils and fats
Dairy products
Processed rice
Sugar
Food products nec
Beverages and tobacco products
Textiles
Wearing apparel
Leather products
Wood products
Paper products; publishing
Petroleum; coal products
Chemical; rubber; plastic products
Mineral products nec
Ferrous metals
Metals nec
Metal products
Motor vehicles and parts
Transport equipment nec
Electronic equipment
Machinery and equipment nec
Manufactures nec
Electricity
Gas manufacture; distribution
Water
Construction
Trade
Transport nec
Water transport
Air transport
Communication
Financial services nec
Insurance
Business services nec
Recreational and other services
Public Administration; Defense; Education; Health
Dwellings
Australia
New Zealand
Rest of Oceania
Hong Kong
Japan
South Korea
Mongolia
Taiwan
Rest of East Asia
Brunei Darussalam
Cambodia
Indonesia
Lao's
Malaysia
Philippines
Singapore
Thailand
Viet Nam
Rest of Southeast Asia
Bangladesh
India
Nepal
Pakistan
Sri Lanka
Rest of South Asia
Canada
United States of America
Mexico
Rest of North America
Argentina
Bolivia
Brazil
Chile
Colombia
Ecuador
Paraguay
Peru
Uruguay
Venezuela
Rest of South America
Costa Rica
Guatemala
Honduras
Nicaragua
Panama
El Salvador
Rest of Central America
Dominican Republic
Jamaica
Puerto Rico
Trinidad and Tobago
Caribbean
Austria
Belgium
Cyprus
Czech Republic
Denmark
Estonia
Finland
France
Germany
Greece
Hungary
Ireland
Italy
Latvia
Lithuania
Luxembourg
Malta
Netherlands
Poland
Portugal
Slovakia
Slovenia
Spain
Sweden
United Kingdom
Switzerland
Norway
Rest of EFTA
Albania
Bulgaria
Belarus
Croatia
Romania
Russian Federation
Ukraine
Rest of Eastern Europe
Rest of Europe
Kazakhstan
Kyrgyzstan
Rest of Former Soviet Union
Armenia
Azerbaijan
Georgia
Bahrain
Iran
Israel
Jordan
Kuwait
Oman
Qatar
Saudi Arabia
Turkey
United Arab Emirates
Rest of Western Asia
Egypt
Morocco
Tunisia
Rest of North Africa
Benin
Burkina Faso
Cameroon
Cote d'Ivoire
Ghana
Guinea
Nigeria
Senegal
Togo
Rest of Western Africa
Central Africa
South Central Africa
Ethiopia
Kenya
Madagascar
Malawi
Mauritius
Mozambique
Rwanda
Tanzania
Uganda
Zambia
Zimbabwe
Rest of Eastern Africa
Botswana
Namibia
South Africa
Rest of South African Customs Union
Rest of the World

(b)

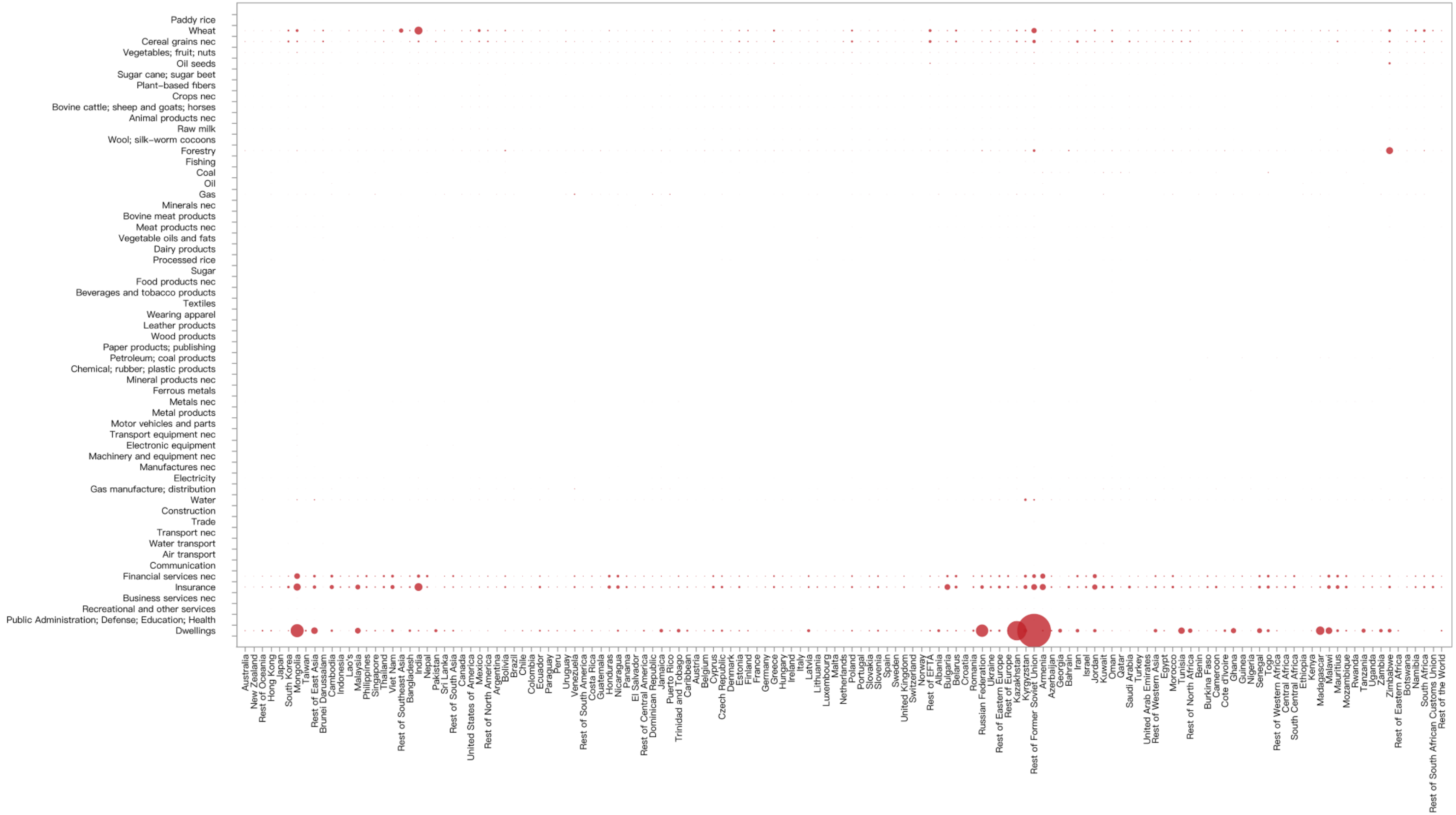
Paddy rice
Wheat
Cereal grains nec
Vegetables; fruit; nuts
Oil seeds
Sugar cane; sugar beet
Plant−based fibers
Crops nec
Bovine cattle; sheep and goats; horses
Animal products nec
Raw milk
Wool; silk−worm cocoons
Forestry
Fishing
Coal
Oil
Gas
Minerals nec
Bovine meat products
Meat products nec
Vegetable oils and fats
Dairy products
Processed rice
Sugar
Food products nec
Beverages and tobacco products
Textiles
Wearing apparel
Leather products
Wood products
Paper products; publishing
Petroleum; coal products
Chemical; rubber; plastic products
Mineral products nec
Ferrous metals
Metals nec
Metal products
Motor vehicles and parts
Transport equipment nec
Electronic equipment
Machinery and equipment nec
Manufactures nec
Electricity
Gas manufacture; distribution
Water
Construction
Trade
Transport nec
Water transport
Air transport
Communication
Financial services nec
Insurance
Business services nec
Recreational and other services
Public Administration; Defense; Education; Health
Dwellings

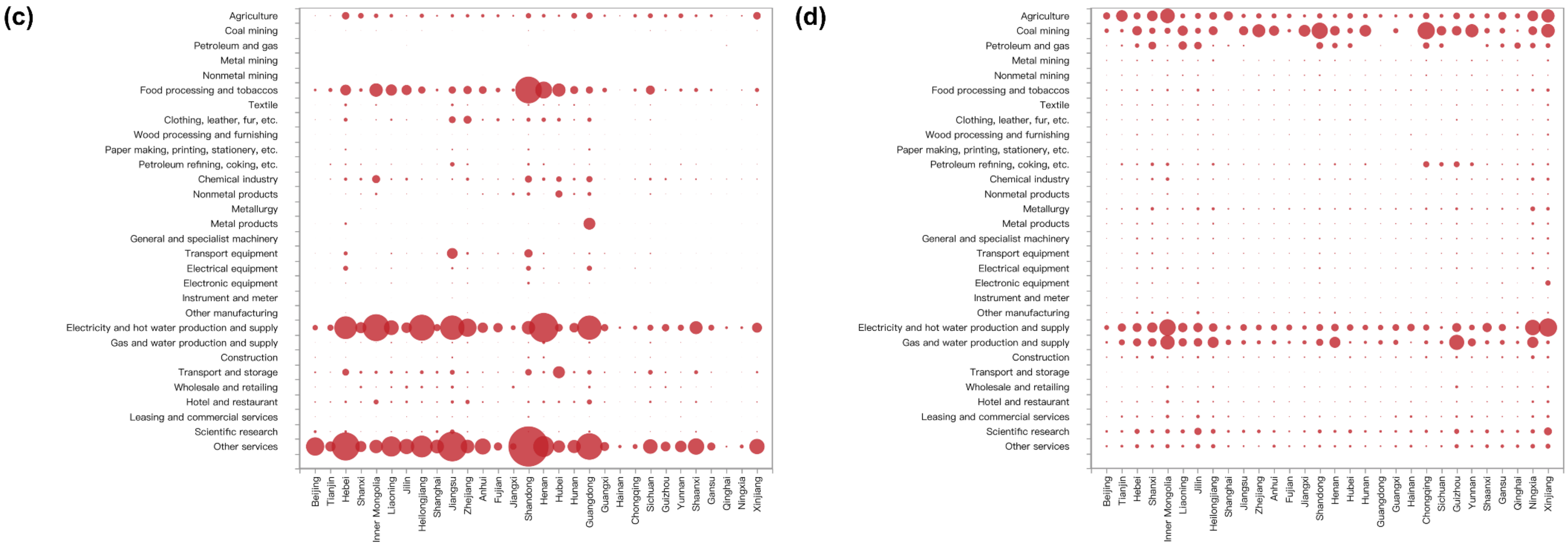

Supplementary Figure 5. Global CO_2 emission reductions and differences in cumulative CO_2 emission intensity of region-sectors in the consummate greener consumption scenario. Supplementary Figures 5a and 5c show global CO_2 emission reductions (Mt) caused by greener consumption in China. Supplementary Figures 5b and 5d illustrate the differences in cumulative CO_2 emission intensity of region-sectors, which equal to original cumulative CO_2 emission intensity of a sector divided by this sector's lowest cumulative CO_2 emission intensity across regions. Larger circles indicate larger values.

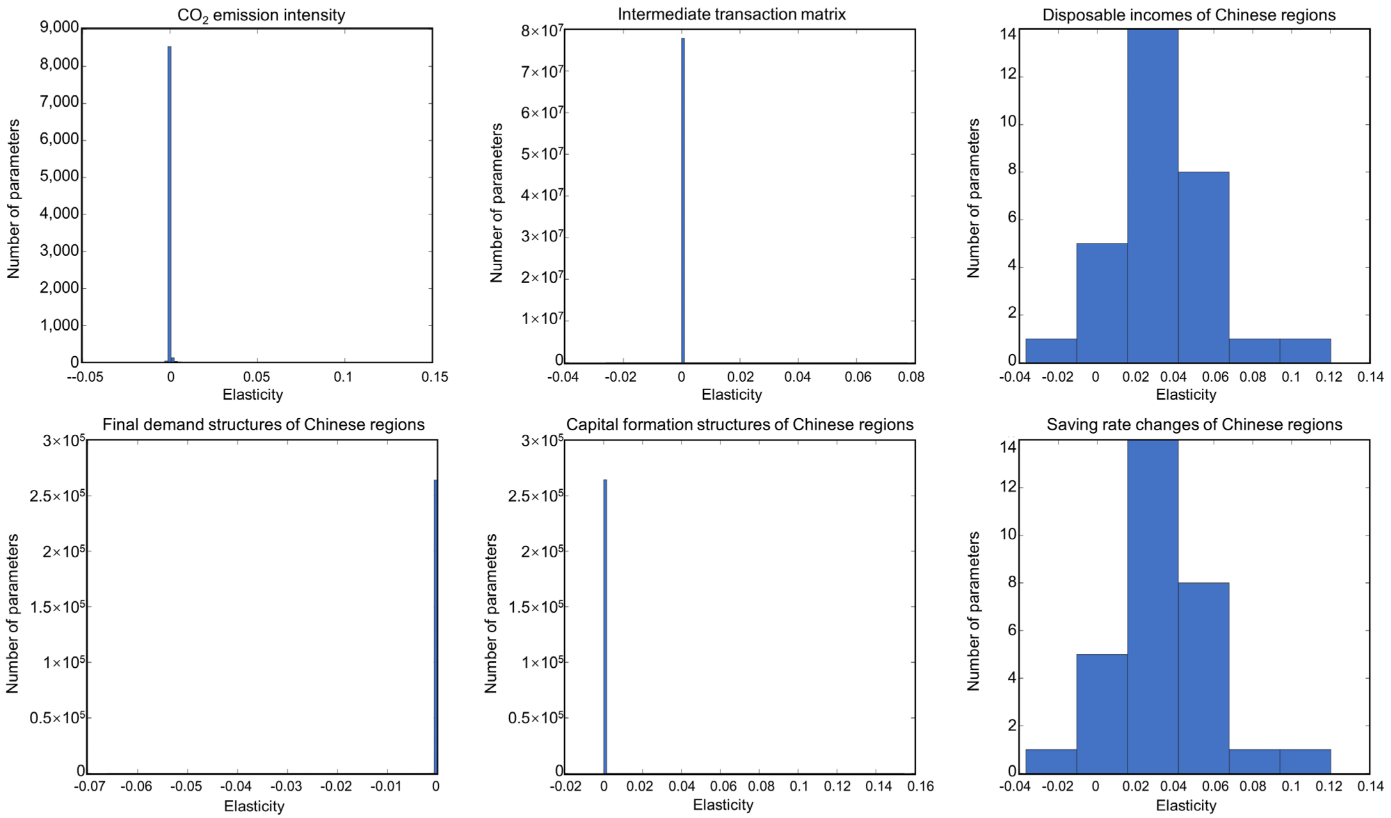

Supplementary Figure 6. Distributions of elasticities for all the parameters of the model developed in this study. The X-axis represent the elasticities of the parameters in CO_2 emission intensity, intermediate transaction matrix, disposable incomes of Chinese regions, final demand and capital formation structures of Chinese regions, and the saving rate changes of Chinese regions. The Y-axis represents the number of the parameters. The bars in the graphs represent the number of parameters whose elasticities are within the range.

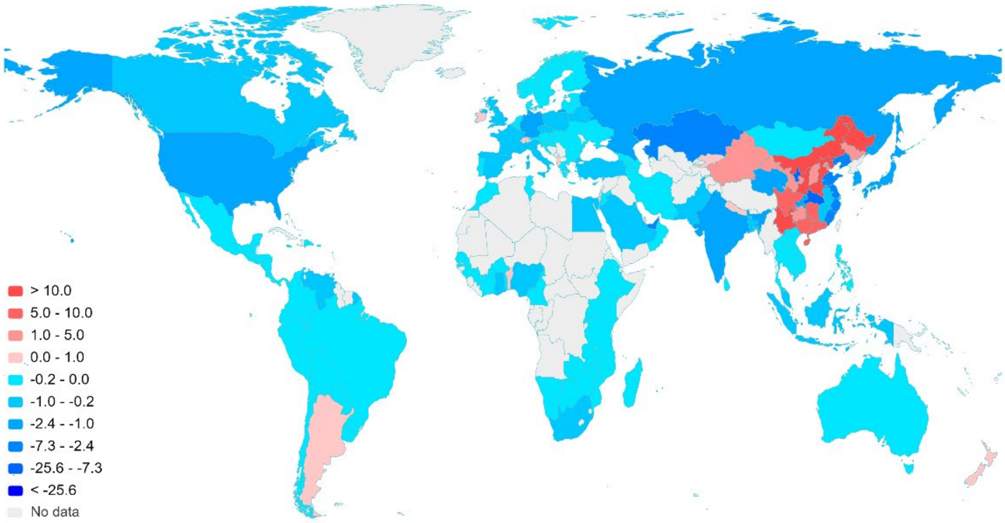

Supplementary Figure 7. Changes in CO_2 emissions of nations and Chinese regions, with conditions of saving rates of all Chinese regions decreasing by 15 percentage points simultaneously, emission intensity adjusted with changes in intermediate inputs, and fixed production levels (Scenario 5).

Supplementary Note 1

Situations with fixed production levels

This study has investigated scenarios with flexible production levels, assuming that an economy can smoothly adjust production to satisfy changes in final demand. In this case, decreases in the saving rates in China can significantly reduce global CO_2 emissions. However, this smoothness of production level adjustment has been challenged by some literature, because the production levels can be fixed due to economic frictions such as sunk costs, firing cost, and government policies[2]. For instance, in China, large firms cannot easily shut down because governments handle employment and tax income issues. To consider the consequences of the frictions mentioned above, we calculate global CO_2 emissions with both reductions in China's saving rate and the assumption of fixed production levels.

In situations with fixed production levels, a 15-percentage-point decrease in the saving rate in China can increase global CO_2 emissions by 155 Mt, including an 18 Mt (0.1%) increase in CO_2 emissions from global production systems and a 137 Mt increase in CO_2 emissions from Chinese households (more results in Supplementary Data 23). The results in Supplementary Figure 7 are significantly different from those in Figure 1 in the main text, which implies that if the production levels are fixed, then the CO_2 emissions of northern and western China will increase. The decrease in capital formation is supposed to decrease global CO_2 emissions. However, if the output levels

remain unchanged, then the saved products will be used (in a sense, wasted) in the intermediate production system. More seriously, the carbon fixed in capital goods will be emitted with the usage of intermediate inputs. For instance, lubricating oil originating from crude oil is part of equipment and machinery, and plastic products originating from crude oil are a part of buildings. The carbon embedded in these chemical products originating from crude oil is fixed in capital goods. A decrease in the capital formation reduces the demand for these chemical products and their upstream crude oil. However, the production capacities of these chemical products and crude oil are still present. If government policies force crude oil production firms to maintain their production scales, then the surplus crude oil will be consumed as intermediate inputs (e.g., producing energy products for energy combustion of other sectors) and generate CO_2 emissions. Thus, with fixed production levels, a decrease in the saving rates will probably release the fixed carbon in capital goods. Fossil fuel reserves are mainly distributed in northern and western China; under this scenario, a decrease in the saving rate will increase CO_2 emissions from northern and western China.

This scenario shows a potential mechanism for why a decrease in the saving rates in China may have a negative effect on global CO_2 mitigation. This finding implies a trade-off relationship between employment guarantees and CO_2 mitigation. If governments try to maintain production levels to stabilise the unemployment rate, then the surplus products will probably generate more CO_2 emissions.

Methods for the estimation of global CO_2 emissions with fixed production levels

In this scenario, we assume that sectoral total outputs remain constant, but global production structures and sectoral CO_2 emission intensity change. The updated global MRIO table is estimated using the RAS method[3,4]. This method adopts the sum of rows and the sum of columns in the total output matrix $\mathbf{x}$ to adjust the direct input coefficient matrix $\mathbf{A}$. The notations $\mathbf{r}$ and $\mathbf{s}$ represent the vectors of parameters for adjusting the sum of rows and the sum of columns, respectively. The calculation is an iterative process that includes the following steps.

(1) We estimate the intermediate input matrix $\mathbf{Z_1}$ with equation (S1):

$$\mathbf{Z_1} = \mathbf{A}\hat{\mathbf{x}} \quad \text{(S1)}$$

The notation $\hat{\mathbf{x}}$ is the diagonal matrix of the total output $\mathbf{x}$. According to the assumption that global total outputs remain constant, the matrix $\mathbf{Z_1}$ equals the original intermediate input matrix $\mathbf{Z}$.

The sum of intermediate outputs and inputs is obtained with equations (S2) and (S3), respectively:

$$\mathbf{u}^* = \mathbf{x} - \mathbf{f}^* \quad \text{(S2)}$$

$$\mathbf{v}^* = \mathbf{x} - \mathbf{v} \quad \text{(S3)}$$

where $\mathbf{f}^*$ is the updated vector of final demand with changed saving rates and $\mathbf{v}$ is a vector of the primary inputs.

(2) We adjust the rows.

The vector of adjustment coefficient $\mathbf{r_1}$ is obtained with equation (S4):

$$r_{1,j} = \frac{u_{1,j}}{u_j^*} \quad \text{(S4)}$$

where $u_{1,j}$ is the sum of row j in the matrix $\mathbf{Z_1}$.

The adjusted intermediate input matrix $\mathbf{Z_2}$ is obtained with equation (S5):

$$\mathbf{Z_2} = \widehat{\mathbf{r_1}}\mathbf{A}\hat{\mathbf{x}} \quad \text{(S5)}$$

(3) We adjust the columns.

The vector of the adjustment coefficient $\mathbf{s_1}$ is obtained with equation (S6):

$$s_{1,j} = \frac{v_{1,j}}{v_j^*} \quad \text{(S6)}$$

where $v_{1,j}$ is the sum of column j in the matrix $\mathbf{Z_1}$.

The adjusted intermediate input matrix $\mathbf{Z_3}$ is obtained with equation (S7):

$$\mathbf{Z_3} = \widehat{\mathbf{r_1}}\mathbf{A}\hat{\mathbf{x}}\widehat{\mathbf{s_1}} \quad \text{(S7)}$$

(4) We compare the targeted vector $\mathbf{u}^*$ with the vector $\mathbf{u_2}$, which is the sum of the rows in the matrix $\mathbf{Z_2}$. If they are equal or close, then the targeted intermediate input matrix is obtained. Otherwise, we repeat steps 2 to 4 until we obtain the targeted intermediate input matrix $\mathbf{Z}^*$, as shown in equation (S8).

$$\mathbf{Z}^* = \cdots \widehat{\mathbf{r_2}}\widehat{\mathbf{r_1}}\mathbf{A}\hat{\mathbf{x}}\widehat{\mathbf{s_1}}\widehat{\mathbf{s_2}} \cdots \quad \text{(S8)}$$

Since the final demand equals that with flexible production levels, the CO_2 emissions from Chinese households with fixed production levels are equal to those with flexible production levels. CO_2 emissions from global production systems with fixed production levels are estimated based on sectoral energy uses. We estimate CO_2 emissions based on the intermediate input matrix of the fixed production level scenario.

(1) The CO_2 emissions for each type of energy source in various sectors **TE** are obtained with equation (S9):

$$\mathbf{TE} = \mathbf{G} * \mathbf{EF} \tag{S9}$$

where $\mathbf{G}$ is a matrix for the uses of each type of energy by sector. The data for sectoral energy uses in Chinese regions are from previous studies[5,6] , while the sectoral energy uses of the other 139 global regions are from the World Energy Statistics (2018 Edition)[7]. The matrix $\mathbf{EF}$ indicates the CO_2 emission factors of energy sources by sector, which are from previous studies[5,8].

The monetary values of energy use by sector $\mathbf{TP}$ are obtained with equation (S10):

$$\mathbf{TP} = \mathbf{G} * \mathbf{P} \tag{S10}$$

where $\mathbf{P}$ is a matrix of energy prices for each type of energy source by sector. The data for energy prices are from the World Energy Prices (2018 Edition)[9].

(2) By matching each type of energy source to the related sectors of the global MRIO model, we can reshape $\mathbf{TE}$ and $\mathbf{TP}$ into new matrixes $\mathbf{TE}'$ and $\mathbf{TP}'$, respectively. Each row represents a sector producing energy products, and each column represents each sector using energy products. The CO_2 emissions of sector *j* for using a unit of value of energy produced in sector *m*, $w_{m,j}$, are obtained with equation (S11):

$$w_{m,j} = \frac{\mathrm{TE}'_{m,j}}{\mathrm{TP}'_{m,j}} \tag{S11}$$

where $\mathrm{TE}'_{m,j}$ is the element of the matrix $\mathbf{TE}'$, representing the CO_2 emissions of sector *j* for using energy produced by sector *m*; and $\mathrm{TP}'_{m,j}$ is the element of the matrix $\mathbf{TP}'$, representing the monetary value from the energy sector *m* to sector *j*.

(3) We calculate the CO_2 emissions of each sector based on its CO_2 emissions per unit of energy value and the intermediate inputs of global energy sectors with equation

(S12).

$$e_j' = \sum_1^m (z_{m,j} * w_{m,j}) \tag{S12}$$

The notation e_j' is the element of the vector $\mathbf{e}'$, representing the CO_2 emissions of sector *j*; $z_{m,j}$ is the element of the matrix **Z**, representing the intermediate input from energy sector *m* to sector *j*.

We assume that there is a linear relationship between e_j' and the CO_2 emissions of sector j in 2012 (e_j). The linear relationship is expressed by the ratio factor r_j, as shown in equation (S13).

$$r_j = \frac{e_j'}{e_j} \tag{S13}$$

The r_j is the element of vector **r**, representing the ratio factor for sector j.

(4) CO_2 emissions from global production systems with fixed production levels are calculated by equation (S14):

$$e_i^{**} = \frac{\sum_1^m (z_{m,i}^* \times w_{m,i})}{r_i} \tag{S14}$$

where e_i^{**} is the element of the vector $\mathbf{e}^{**}$, representing the CO_2 emissions of sector *i* from global production systems with fixed production levels; $z_{m,i}^*$ is the element of the matrix $\mathbf{Z}^*$, representing the updated intermediate input from energy sector *m* to sector *i*.

Data availability

The data regarding the estimations of CO_2 emissions with fixed production levels also include sectoral energy uses, energy prices, and the CO_2 emission factors of each

type of energy source. The sectoral energy uses of Chinese regions are from previous studies[5,6], while the energy uses of nations other than China can be obtained from the World Energy Statistics (2018 Edition) published by the International Energy Agency (IEA) (https://www.iea.org/subscribe-to-data-services/world-energy-balances-and-statistics)[7]. The prices of energy products are from the World Energy Prices (2018 Edition) published by the IEA (https://www.iea.org/subscribe-to-data-services/prices-and-taxes)[9]. The CO_2 emission factors of various energy sources for Chinese regions are from previous studies[5,8], while those for nations other than China are from the Intergovernmental Panel on Climate Change (IPCC) (https://www.ipcc.ch/report/2006-ipcc-guidelines-for-national-greenhouse-gas-inventories/)[10].

Supplementary References

1 National Bureau of Statistics. Annual Data of China. http://data.stats.gov.cn/easyquery.htm?cn=C01 (2020).

2 Moscoso Boedo, H. J. & Mukoyama, T. Evaluating the effects of entry regulations and firing costs on international income differences. J. Econ. Growth 17, 143-170 (2012).

3 Miller, R. E. Input-output Analysis Foundations and Extensions. 2nd edn, (Cambridge University Press, 2009).

4 Timmer, M. P., Dietzenbacher, E., Los, B., Stehrer, R. & de Vries, G. J. An Illustrated User Guide to the World Input–Output Database: the Case of Global Automotive Production. Rev. Int. Econ. 23, 575-605 (2015).

5 Shan, Y. et al. China CO_2 emission accounts 1997–2015. Sci. Data 5, 170201 (2018).

6 Shan, Y. et al. New provincial CO_2 emission inventories in China based on apparent energy consumption data and updated emission factors. Appl. Energy 184, 742-750 (2016).

7 International Energy Agency. World Energy Statistics 2018 Edition. (International Energy Agency, Paris, 2018).

8 Liu, Z. et al. Reduced carbon emission estimates from fossil fuel combustion and cement production in China. Nature 524, 335 (2015).

9 International Energy Agency. World Energy Prices 2018 Edition. (International Energy Agency, Paris, 2018).

10 Intergovernmental Panel on Climate Change (IPCC). 2006 IPCC Guidelines for National Greenhouse Gas Inventories. (IPCC, Kanagawa, Japan, 2006).
