# Supplementary material for: Saving less in China facilitates global CO2 mitigation
Source: Nat Commun. 2020 Mar 13;11:1358. doi: 10.1038/s41467-020-15175-2 (PMC7070072; doi:10.1038/s41467-020-15175-2)
Supplement: Supplementary file 4 — Description of Additional Supplementary Files [file 41467_2020_15175_MOESM4_ESM.pdf]

Description of Additional Supplementary Files

File Name: Supplementary Data 1
Description: CO_2 emissions with flexible production levels (Mt)

File Name: Supplementary Data 2
Description: Global CO_2 emissions induced by unitary final consumption in China - sectors of nations other than China (Mt)

File Name: Supplementary Data 3
Description: Global CO_2 emissions induced by unitary final consumption in China - sectors of Chinese regions (Mt)

File Name: Supplementary Data 4
Description: Global CO_2 emissions induced by unitary capital formation in China - sectors of nations other than China (Mt)

File Name: Supplementary Data 5
Description: Global CO_2 emissions induced by unitary capital formation in China - sectors of Chinese regions (Mt)

File Name: Supplementary Data 6
Description: Global CO_2 emission changes caused by reducing the saving rate of each Chinese region by 15 percentage points (Mt)

File Name: Supplementary Data 7
Description: Sectoral structure of global CO_2 emission changes caused by the decrease of saving rates - case of Shandong

File Name: Supplementary Data 8
Description: Sectoral structure of global CO_2 emission changes caused by the decrease of saving rates - case of Inner Mongolia

File Name: Supplementary Data 9
Description: Global CO_2 emission changes caused by reducing the saving rate of each Chinese region to saving rate of Shanghai in 2012 (Mt)

File Name: Supplementary Data 10
Description: Uncertainties of global CO_2 emissions with flexible production levels (Mt)

File Name: Supplementary Data 11
Description: Sensitivity coefficients and elasticities for CO_2 emission intensity - global industrial CO_2 emissions

File Name: Supplementary Data 12
Description: Sensitivity coefficients for intermediate transaction matrix (taking China’s domestic intermediate transaction matrix for example) - global industrial CO_2 emissions.

File Name: Supplementary Data 13
Description: Elasticities for intermediate transaction matrix (taking China’s domestic intermediate transaction matrix for example) - global industrial CO_2 emissions.

File Name: Supplementary Data 14
Description: Sensitivity coefficients and elasticities for disposable incomes of Chinese regions - global industrial CO_2 emissions

File Name: Supplementary Data 15
Description: Sensitivity coefficients for final consumption structures of Chinese regions - global industrial CO_2 emissions

File Name: Supplementary Data 16
Description: Elasticities for final consumption structures of Chinese regions - global industrial CO_2 emissions

File Name: Supplementary Data 17
Description: Sensitivity coefficients for capital formation structures of Chinese regions - global industrial CO_2 emissions

File Name: Supplementary Data 18
Description: Elasticities for capital formation structures of Chinese regions - global industrial CO_2 emissions

File Name: Supplementary Data 19
Description: Sensitivity coefficients and elasticities for saving rate changes of China - global industrial CO_2 emissions

File Name: Supplementary Data 20
Description: Sensitivity coefficients and elasticities for saving rate changes of China - Chinese household emissions

File Name: Supplementary Data 21
Description: The list of nations and Chinese regions in the global MRIO table

File Name: Supplementary Data 22
Description: Sector classification of the global MRIO table

File Name: Supplementary Data 23
Description: Global CO_2 emissions with fixed production levels (Mt)
